# Granular cell tumor of the breast: a case report

**DOI:** 10.1186/1752-1947-8-465

**Published:** 2014-12-26

**Authors:** Nawal Hammas, Hind El Fatemi, Sofia Jayi, Imane Hafid, Ghizlane Fikri, Aziza El Houari, Nadia Seqqali, Siham Tizniti, Moulay Abdelilah Melhouf, Afaf Amarti

**Affiliations:** 34 Department of Pathology, Hassan II University Hospital, Fez, Morocco; 35 Department of Obstetrics and Gynecology II, Hassan II University Hospital, Fez, Morocco; 36 Department of Radiology, Hassan II University Hospital, Fez, Morocco; 37 Appartement N°4, Résidence Majdouline, Route Sidi Hrazem, Bel Air, Fès Morocco

**Keywords:** Breast, Granular cells tumor, Schwann cells, S-100 protein

## Abstract

**Introduction:**

A granular cell tumor involving the breast parenchyma was first described by Abrikossoff in 1931. Localization of this lesion to the breast is very rare, accounting for between 5% and 15% of all granular cell tumor cases. We present this case because of the rarity of this tumor. It is frequently confused with breast carcinoma on clinical and radiological examination, and its diagnosis can therefore be challenging for clinicians, radiologists and pathologists.

**Case presentation:**

We report the case of a 32-year-old Moroccan woman who presented with a palpable mass in her right breast. Mammography and ultrasound examination revealed a heterogeneous, irregular and poorly limited mass, located at the union of the outer quadrants of her right breast. The mass was in contact with her latissimus dorsi and suspicious for malignancy. A histological examination combined with immunohistochemical study revealed it to be a granular cell tumor.

**Conclusion:**

Although a granular cell tumor of the breast is a rare breast neoplasm, it should be considered in the differential diagnosis of benign and malignant lesions. Pathologists should bear in mind a granular cell tumor when examining material containing cells with abundant granular cytoplasm to avoid misdiagnosing breast carcinoma, which could lead to unnecessary surgery.

## Introduction

A granular cell tumor (GCT) is an uncommon neoplasm that was first alluded to by Weber in 1854. It was fully described by Abrikossoff in 1926, who suspected a myogenic origin and therefore termed it a granular cell myoblastoma. However, because of S-100 protein positivity and the similarity of the tumor cells to Schwann cells, researchers proposed that the tumor originated from the Schwann cells; the exact histogenesis of this tumor is still unknown [1–4]. It typically arises in the tongue but it may occur at any site and at any age, and can be multifocal. A GCT involving the breast parenchyma was first described by Abrikossoff in 1931 [2]. Localization of this lesion to the breast is very rare, accounting for between 5% and 15% of all GCT cases [1, 2]. Although GCT is a well-established entity, it is frequently confused on clinical and radiological examination with breast carcinoma. Its diagnosis may be a challenge for clinicians, radiologists and pathologists [1, 5].

We report a case of a GCT of the breast mimicking carcinoma on mammography and ultrasonography. The diagnosis was made by histological examination. Through this observation, we discuss the radio-clinical, histopathological and therapeutic aspects of this rare tumor, as well as outcomes.

## Case presentation

We report the case of a 32-year-old Moroccan woman who presented with a palpable mass in her right breast of two year’s duration. She had no personal or family history of malignancy. A physical examination showed a 1.5cm firm, painless mass located at the union of the outer quadrants of her right breast, without any alterations to her skin or axillary lymph nodes.

**Figure 1:**
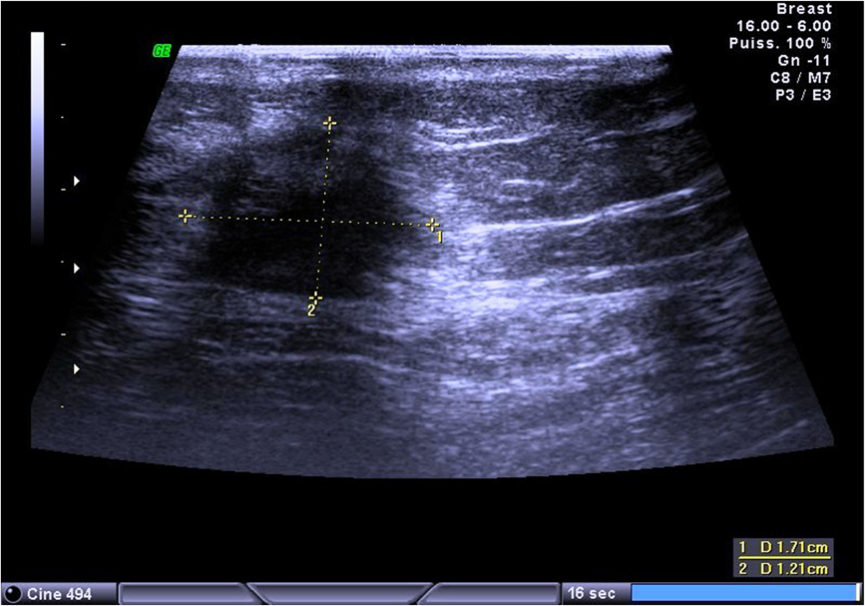
**Ultrasound demonstrated a 17mm hypoechoic, heterogeneous, irregular and poorly limited mass, suspicious for malignancy.**

A mammogram revealed a dense mass with ill-defined borders. An ultrasound demonstrated a 17mm hypoechoic, heterogeneous, irregular and poorly limited mass, located at the union of the outer quadrants of her right breast, in contact with her latissimus dorsi. The mass was suspicious for malignancy (Figure 1).

**Figure 2:**
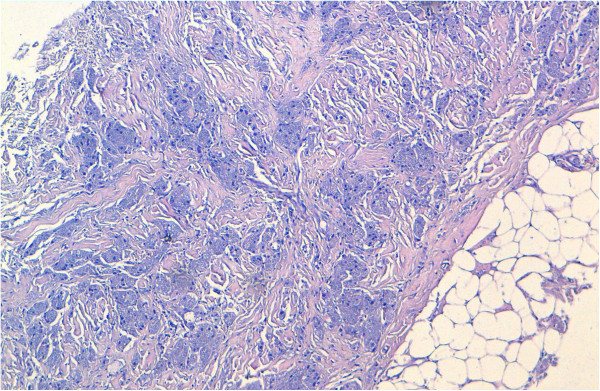
**Microscopic appearance: the tumor was composed of compact nests of polygonal cells with well-defined cell borders. Hematoxylin and eosin stain; original magnification ×100.**

**Figure 3:**
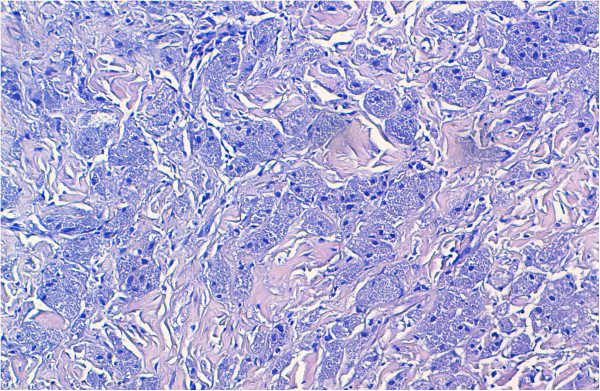
**Microscopic appearance: tumor cells contained granular eosinophilic cytoplasm, and small, uniform, round nuclei without nuclear pleomorphism or mitotic activity. Hematoxylin and eosin; original magnification ×200.**

**Figure 4:**
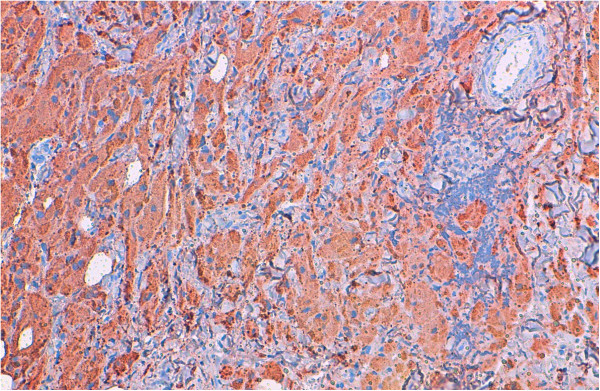
**Immunohistochemical study showed reactivity for S-100 protein. Original magnification ×100.**

On gross examination, the tumor was 2cm at its greatest diameter, whitish and had ill-defined borders. Microscopic examination revealed a benign tumor composed of compact nests of polygonal cells with well-defined cell borders that contained granular eosinophilic cytoplasm, and small, uniform, round nuclei without nuclear pleomorphism or mitotic activity (Figures 2 and 3). An immunohistochemical analysis showed positive staining for S-100 protein (Figure 4). The cells were negative for cytokeratins and cluster of differentiation (CD) 163. Based on these data, the diagnosis of GCT was confirmed.

## Discussion

A GCT is an uncommon tumor that may arise throughout the body. The most common anatomical organ site of origin is the tongue, followed by soft tissues [1, 4, 6]. GCT of the breast accounts for between 5% and 15% of all GCT cases [2]. It occurs in a wide range of ages, from teenagers to the elderly, most commonly in women between 30 and 50 years of age, and especially in women of African-American origin. However, some cases of GCTs of the breast have been described in men [1, 4, 6].

This tumor usually presents as a firm and painless mass, usually well circumscribed, and generally mobile. Several cases have been reported with poorly circumscribed masses that may be fixed to the pectoral muscle, mimicking a malignant lesion [1, 2]. Skin involvement, including thickening, tethering, dimpling and retraction, has been described [2]. Usually the tumor is solitary, but multiple (multifocal) lesions have been reported in 5.4% to 17.6% of cases [4, 6]. In our patient, the tumor was poorly limited and was in contact with her latissimus dorsi.

GCTs of the breast arise from intra-lobular breast stroma. They display no side preference [2, 6, 7]. They occur more frequently in the upper inner quadrant of the breast, in contrast to breast carcinoma, which is more usually located in the upper outer quadrant. This reflects the course of the supraclavicular nerve, which innervates the breast skin. However, a case analysis elicited a wide variety of locations, including the upper outer quadrant, the upper inner quadrant, the axillary tail, the midline, the nipple and the subareolar region [1, 2].

The histogenesis of GCT remains controversial and its etiology undiscovered. When Abrikossoff first described the tumour type he postulated that they originate in the skeletal muscle [2]. Chung and Work went on to suggest a smooth muscle origin [8]. Then it was thought that they derive from fibroblastic or undifferentiated mesenchymal or histiocytic cells and Ulrich et al. showing evidence of histiocytic origin [9]. Additionally, immunohistochemical profiling suggests that they are unlikely to be of muscle (because of negativity for alpha-smooth muscle actin) or epithelial (because of negativity for keratin or epithelial membrane antigen) origin [10]. Later, researchers proposed that the tumors originate from the Schwann cells because of their S-100 protein positivity and the similarity of the tumor cells to Schwann cells [3].

The presentation of GCTs of the breast on diagnostic imaging is variable. On mammography, it has often been described as a small (<3cm) lesion, ranging from a round, well-circumscribed mass, to an indistinct or spiculated lesion lacking calcifications, difficult to distinguish from carcinoma [4–6]. On ultrasound, it can present as a solid, poorly marginated lesion, with marked posterior shadowing, suggestive of carcinoma, or as a more benign-appearing well-circumscribed solid mass [1, 4, 6].

A gross section usually shows a firm or hard, homogenous, grayish-white to yellow tumor, measuring generally 3cm or smaller, but tumors measuring up to 6cm have been reported. Most of the tumors appear to be well circumscribed, but other examples have ill-defined borders and may infiltrate, as in our case, into the surrounding tissues, particularly fibrous tissue, adipose tissue and the pectoralis major muscle. These features mimic malignant growth patterns and give the impression of scirrhous carcinoma [1, 2, 6, 7].

On clinical and radiographic examination, it is impossible to establish a definitive diagnosis of GCT of the breast without a biopsy. Sonographically guided percutaneous biopsy of the lesion is well established as the diagnostic procedure of choice for histopathology sampling [7]. On microscopy, the tumor is well circumscribed but may have infiltrative margins, as noted in our case. The cells are arranged in nests and sheets. They are generally uniform, large, bland and polygonal. However, rarely they may be round or spindle-like in shape [1, 2]. They have distinct borders and abundant granular eosinophilic cytoplasm, from which this tumor derives its name. The granular change is caused by cytoplasmic accumulation of lysosomes. Nuclei are small, centrally located and hyperchromatic with one or two nucleoli. They do not display mitoses, pleomorphism, nuclear multiplicity or atypia [1, 2, 4, 7]. Multi-nucleation and rare mitotic features may be seen, but these features should not be interpreted as evidence of malignancy. Variable amounts of collagenous stroma are present. Histochemical analysis confirms whether the granules are diastase resistant and Periodic acid-Schiff positive [1, 6]. The definitive diagnosis of GCT is only possible with immunohistochemical examination. The tumor cells are strongly immunoreactive to S-100 protein. They will not show staining for cytokeratins, epithelial membrane antigen or mucin. The cells were reported to be positive for CD68, carcinoembryonic antigen and vimentin in some cases in the literature [1, 3]. In our presented case, the description of the pathological features was supported by immunohistochemistry: S-100 protein positivity and cytokeratin negativity.

While the majority of GCTs behave in a benign manner, occasional malignant cases have been described (less than 1% of all GCTs, including mammary lesions, are malignant) [4, 6, 7]. The distinction between benign and malignant GCTs was proposed by Le et al [11] and Adeniran *et al.* [7], and included the criteria of necrosis, spindling, vesicular nuclei with large nucleoli, increased mitotic activity (more than two mitoses per 10 high power field at ×200 magnification), high nuclear to cytoplasmic ratio, and nuclear pleomorphism. These criteria classify GCT by histology into atypical (when two of these six criteria are present) and malignant (when three or more of these six criteria are met) [4].

GCTs should be distinguished from mammary carcinoma, particularly scirrhous carcinoma and apocrine carcinoma. The difference between GCT and granulomatous inflammatory reaction or a histiocytic tumor is negativity for histiocyte-associated antigens, although reactivity for CD68 has been described in a GCT [12]. GCTs must be distinguished from metastatic neoplasms of the breast that have oncocytic or clear cell features, such as renal carcinoma, malignant melanoma and alveolar soft part sarcoma [1, 6].

Wide local excision with free margins is the treatment of choice. Subtotal excision may lead to local recurrences. Direct invasion of an axillary lymph node by a GCT of the breast that arose in the axillary tail has been reported [1, 3, 6].

## Conclusion

This case illustrates that, although GCT of the breast is a relatively rare breast neoplasm, it should be considered in the differential diagnosis of benign and malignant lesions. Pathologist should bear in mind GCTs when examining material containing cells with abundant granular cytoplasm to avoid misdiagnosing breast carcinoma, which could lead to unnecessary surgery.

## Consent

Written informed consent was obtained from the patient for publication of this case report and any accompanying images. A copy of the written consent is available for review by the Editor-in-Chief of this journal.

## Abbreviations

GCT: granular cell tumor.
